# Rationale and design of a randomized controlled trial examining oral administration of bisphenol A on hepatic glucose production and skeletal muscle insulin sensitivity in adults

**DOI:** 10.1016/j.conctc.2020.100549

**Published:** 2020-02-25

**Authors:** Todd A. Hagobian, Hannah Brunner-Gaydos, Adam Seal, Andrew Schaffner, Chris Kitts, Ryan Hubbard, Steven K. Malin, Michael R. La Frano, Kelly A. Bennion, Suzanne Phelan

**Affiliations:** a Center for Health Research, California Polytechnic State University, USA; b Department of Kinesiology and Public Health, California Polytechnic State University, USA; c Department of Statistics, California Polytechnic State University, USA; d Department of Biology, California Polytechnic State University, USA; e Campus Health and Wellbeing, California Polytechnic State University, USA; f Department of Kinesiology, University of Virginia, USA; g Department of Food Science and Nutrition, California Polytechnic State University, USA; h Department of Psychology and Child Development, California Polytechnic State University, USA

**Keywords:** Bisphenol A, Administration, Diabetes, Insulin sensitivity, Glucose

## Abstract

Previous observational studies have shown that the endocrine disrupting chemical bisphenol A (BPA) is associated with type 2 diabetes, but few studies have examined direct effects of BPA on human health. The purpose of this study is to determine whether orally administered BPA at the US Environmental Protection Agency (EPA) safe dose of 50 μg/kg body weight has an adverse effect on hepatic glucose production and skeletal muscle insulin sensitivity. Forty, non-habitually active, healthy adults of normal weight will be enrolled. Participants will begin with a 2-day baseline energy balance diet low in bisphenols in which urine and blood will be collected, and standard tests performed to assess the primary outcome measures of hepatic glucose production (via [6,6-^2^H] glucose infusion) and skeletal muscle insulin sensitivity (via euglycemic hyperinsulinemic clamp technique). Secondary outcome measures are fasting hormones/endocrine factors (insulin, glucose, C-peptide, Pro-insulin, adiponectin, 17-beta-estradiol, free fatty acids) related to the pathogenesis of type 2 diabetes. Participants will then be randomly assigned to a 4-day energy balance diet plus oral administration of BPA at 50 μg/kg body weight (Diet + BPA) or 4-day energy balance diet plus oral administration of placebo (Diet + No BPA); all outcome measures will be reassessed after 4 days. Findings from this study will provide a framework for other studies in this area, and provide much needed experimental evidence using gold standard measures as to whether oral BPA administration over several days poses any risk of type 2 diabetes.

## Highlights

• Bisphenol A is associated with type 2 diabetes.
• Few studies have examined oral bisphenol A administration on the pathogenesis of type 2 diabetes.
• This study will examine oral bisphenol A administration on hepatic glucose [6,6-^2^H] suppression.
• This study will examine insulin sensitivity (euglycemic hyperinsulinemic clamp technique).

## 1 Introduction

The prevalence of diabetes is well established, affecting >29 million Americans, with 90–95% of these individuals diagnosed with type 2 diabetes [1,2]. Diet, physical activity, obesity, and genetics play important roles in the etiology of type 2 diabetes. However as those established factors only explain only 30–60% of variance [3], as much still remains unknown. Emerging data suggest that synthetic non-persistent endocrine disruptors used in a variety of common consumer goods, including the industry-produced chemical BPA, may increase the risk of type 2 diabetes [[4], [5], [6], [7], [8], [9], [10], [11], [12], [13]]. National Health and Nutrition Examination Survey (NHANES), Nurses’ Health Study II (NHSII), and other cross-sectional data have shown associations between urinary BPA concentrations and type 2 diabetes [14,15], pre-diabetes [16], insulin resistance [17], and hemoglobin A1c [18]. The mechanisms linking BPA exposure to diabetes risk remain unclear. Animal and *in vitro* data suggest that BPA has estrogenic activity [5] and disrupts several systems related to the pathogenesis of type 2 diabetes including decreased insulin sensitivity [19], dysregulation of glucose metabolism [20], altered pancreatic *beta* cell and hepatic cell functioning [12,20] and adiponectin release [21].

We recently conducted one of the only known studies in humans that found that oral administration of BPA at the US EPA safe dose of 50 μg/kg body [22] weight immediately decreased blood glucose, insulin, and C-peptide concentrations in response to an oral glucose tolerance test over 3 h [23]. Stahlut et al. similarly showed that BPA administration at the US EPA safe dose immediately decreased insulin and C-peptide concentrations in response to glucose infusion [24]. Although potential mechanisms were not examined, these human data suggest that oral BPA administration may negatively impact hepatic glucose production, skeletal muscle insulin sensitivity, and/or glucose absorption through the gastrointestinal tract and potentially fecal microbiome. These human studies were consistent with the one animal study in mice showing that acute oral BPA consumption at 10 μg/kg body weight significantly reduced glycemia [4]. Interestingly, in the same study, 4 days of BPA administration at 100 μg/kg body weight drastically increased glycemia and the mice became hyperinsulinemic [4]. Taken together, these data suggest that oral administration of BPA may immediately decrease glucose concentrations but may then sharply increase glucose concentrations above baseline by ~20% over several ensuing days. To accurately determine the direct effects of BPA on the progression to type 2 diabetes in humans, well-controlled experimental designs over several days using gold standard measures are required. The primary purpose of this experimental study is to determine the effects of oral administration of BPA over 4 days on hepatic glucose production and skeletal muscle insulin sensitivity in adults using gold standard measures.

## 2 Methods

### 2.1 Overview and outcome measures

**Fig. 1:**
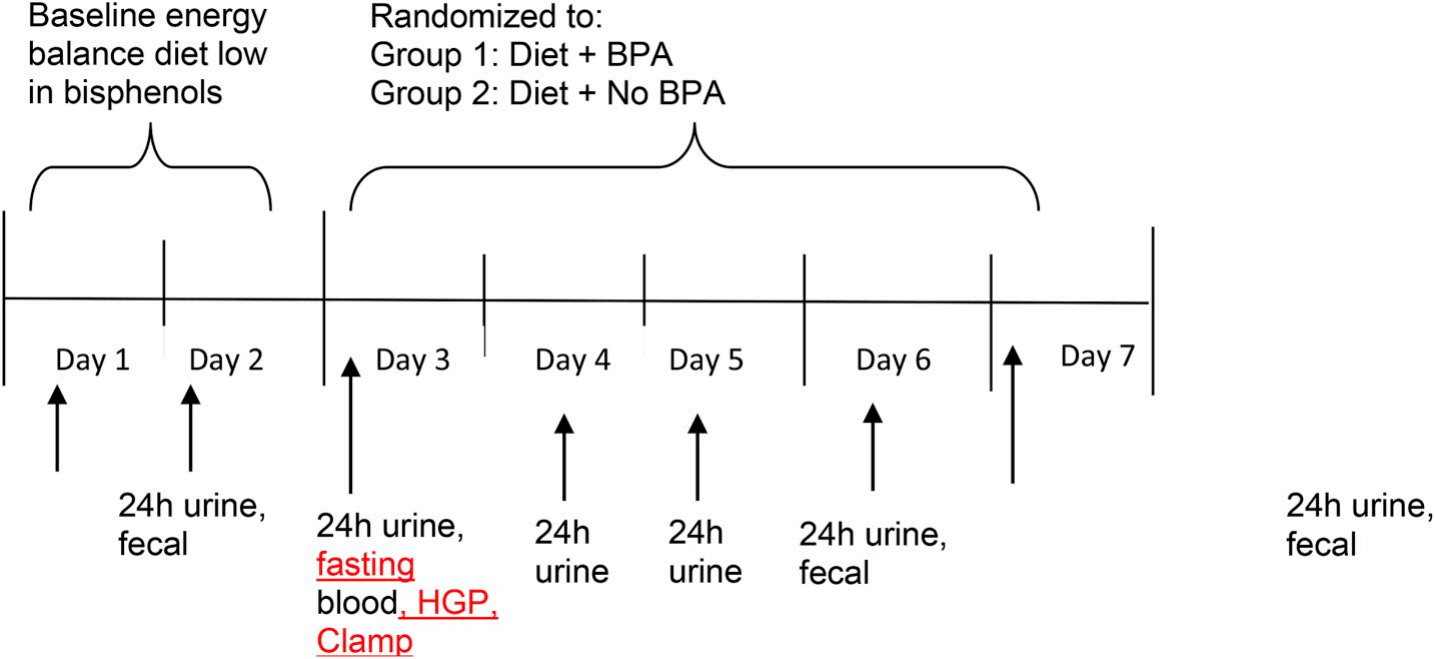
Overview of experimental study design. Participants will reside in our laboratory facilities during which energy intake, energy expenditure and sleep will be monitored and controlled. Urine, blood and fecal samples will be collected using standard methods to assess BPA, hepatic glucose production ([6,6-^2^H] glucose infusion), skeletal muscle insulin sensitivity (euglycemic hyperinsulinemic clamp technique), and the fecal microbiome. HGP, hepatic glucose production; Clamp, skeletal muscle insulin sensitivity.

Fig. 1 provides an overview of the study design. The primary purpose of this 2-group, randomized, double-blinded, experimental study is to determine whether oral administration of BPA at a dose consistent with the US EPA safe dose [22], while controlling for energy intake and energy expenditure, has an independent effect on hepatic glucose production and skeletal muscle insulin sensitivity. Participants will be randomly assigned to a 4-day energy balance diet plus oral administration of BPA at 50 μg/kg body weight (Diet + BPA) or 4-day energy balance diet plus oral administration of placebo (Diet + No BPA). The primary outcome measures are hepatic glucose production via 6,6-^2^H glucose infusion and skeletal muscle insulin sensitivity via euglycemic hyperinsulinemic clamp technique. Secondary outcome measures are fasting hormone and endocrine factors (insulin, glucose, C-peptide, Pro-insulin, adiponectin, 17-beta-estradiol, free fatty acids) related to the pathogenesis of type 2 diabetes, and exploratory outcome is fecal microbiome. This clinical study has established a Data Safety Monitoring Board (DSMB) to review participant safety that includes all senior investigators (Drs. Hagobian, Schaffner, Kitts, Malin, Bennion, La Frano, Phelan) and medical doctor specializing in internal medicine (Dr. Hubbard). The DSMB will formally meet bi-annually, and more frequently if needed.

### 2.2 Participants

Forty, 18–45 years old, non-dieting adults of normal-weight (18.5–24.9 kg/m^2^ BMI), distributed equally between sexes, will be recruited from California Polytechnic State University and the surrounding area in San Luis Obispo, CA. All participants will be healthy, weight-stable for the previous 6 months (no greater than 5 kg gain or loss), free of any metabolic or chronic disease, English speaking, non-smoking, and not habitually active (≤3 h/week of aerobic exercise), assessed by Health and Fitness History as well as Physical Activity Readiness (PAR-Q) questionnaires. We chose participants of normal-weight to minimize the potential confound of high BPA exposure and obesity-mediated insulin resistance [8,[25], [26], [27]]. Both men and women will be included in the study, as previous reports highlight that total BPA exposure does not differ by sex [28]. Exclusion criteria include: Any metabolic or chronic disease including impaired glucose tolerance or type 1 or type 2 diabetes, colitis or any inflammatory bowel condition, neurologic or psychiatric conditions, smoking, unsafe dieting practices, special diets (e.g. vegetarian, low-carbohydrate, Paleolithic, etc.), pregnant women or women trying to become pregnant, and postmenopausal women. All women will be given a pregnancy test (First Response, Princeton, NJ) that detects urine human chorionic gonadotropin to ensure non-pregnant status prior to their participation. All testing in women will start in the early follicular phase (1–4 days after start of menstruation) of the menstrual cycle. All race/ethnicities will be eligible for this study with a target enrollment of 20% Hispanic and 80% non-Hispanic. Because participants will undergo a 2-day baseline run-in with a diet low in BPA, which has been shown to reduce BPA by 66% [29], background BPA exposure is not an exclusion. The Institutional Review Board at California Polytechnic State University has approved the study (project number 2018–149), and verbal and written consent will be obtained from all participants. This study will be carried out in accordance with The Code of Ethics of the World Medical Association (Declaration of Helsinki).

### 2.3 Recruitment and retention

Recruitment will take place via fliers, radio advertisements, and direct solicitation at California Polytechnic State University and the surrounding community in San Luis Obispo, CA. Based on prior research, we expect >98% retention [23,[30], [31], [32], [33], [34]]. Participants will receive a financial incentive of $500 for completing all visits ($100 for baseline measures, $400 after treatment period).

### 2.4 Sequence of events

Preliminary tests include completing a sociodemographic questionnaire assessing age, race, ethnicity, income, employment, education, and weight history. Height will be measured by stadiometer (Ellard Instrumentation LTD., Monroe, WA) and weight in duplicate by balance scale (Continental Scale Corporation, Bridgeview, IL), and BMI (kg/m^2^) will be calculated. Resting metabolic rate (RMR) will be measured in the morning after an overnight fast using a ventilated hood and indirect calorimetry to estimate energy requirements consistent with best practice methods [35]. After a 60-min period of relaxation, participants will sit in a reclining chair for 30–60 min while expired air is collected using the metabolic measurement system (Parvomedics TrueMax 2400, Sandy, UT). Participants will complete a 3-day (2 weekdays, 1 weekend day) dietary recall using the automated NCI ASA-24 (https://epi.grants.cancer.gov/asa24/). Total energy requirements will be estimated using RMR and the appropriate activity factor and 24 h dietary recalls, as others and we previously described [30,33,36,37]. Kien and Ugrasbul [37] reported that energy requirements estimated from RMR and the appropriate activity factor were strongly correlated (r = 0.73) with energy requirements measured during 28-days of controlled feeding. Body composition will be determined with dual-energy X-ray absorptiometry using a Lunar iDXA scan (General Electric Healthcare Company, USA) for descriptive characteristics. Briefly, participants will lay flat on their back without moving for approximately 10 min while a scan arm emitting a low-grade x-ray scans the participants body. Body density and whole-body composition will be determined from the scan.

After preliminary tests, participants will begin with a 2-day run-in baseline energy balance diet low in bisphenols (Fig. 1) in which 24 h-urine, blood, and fecal samples will be collected using standardized tests to assesses skeletal muscle insulin sensitivity (via euglycemic hyperinsulinemic clamp technique), hepatic glucose production (via [6,6-^2^H] glucose infusion), and fecal microbiome. Participants will reside in Cal Poly's sleep laboratory facilities and Center for Health Research during which energy intake, energy expenditure, and sleep will be closely monitored and controlled. Participants will be provided all food, wear an Actigraph GT3X accelerometer on their non-dominant hip for the entire 6 days, and ActiPal on their dominant leg (to assess sitting and standing), and perform no physical activity beyond daily living [38]. Participants will be allowed to leave our facilities and attend work or school but will be required to wear the Actigraph and ActiPal to ensure their typical activity levels. Participants will consume breakfast at our facilities and must return each night by dinnertime to eat and sleep in our facilities. During the day, participants will be provided two snacks and lunch. Participants will be provided an energy balance diet low in BPA and bisphenol S (BPS; <0.20 ng/g fresh weight; e.g. organic, whole foods, etc.). Based on previous studies that have categorized foods to be high and low in BPA [39], we pretested in our laboratory foods from local grocery stores in San Luis Obispo, CA that are low in BPA and BPS using a commercially available enzyme-linked immunosorbent assay (ELISA) kit (Detroit R&D, Inc., Detroit MI; Table 1). BPS, now a common industry substitute for BPA, is also being monitored due to its similar endocrine disruptor effects as BPA including high estrogenic and androgenic activity [40], and association with type 2 diabetes [41]. The composition of the diet will be approximately 55% carbohydrate, 30% fat, and 15% protein, consisting of all natural, organic foods, and all food will be prepared and stored in BPA-free containers, glass containers, etc.

**Table 1:** BPA and BPS concentrations in food samples.

Food	Container Type	Volume (mL)	Weight (g)	BPA (ng/g)	BPS (ng/g)
ORGANIC FOODS					
Cream cheese	Aluminum	237	227	0.006	0.009
41% chocolate	Aluminum	83	80	0.013	0.015
Chile beans	Can	435	416	0.005	0.006
Ravioli	Can	444	425	0.004	0.006
Black bean	Can (BPA free lining)	444	425	0.003	0.006
Pinto beans	Can (BPA Free lining)	444	425	0.004	0.006
Coffee	Cardboard (Aluminum lined)	355	339	ND	0.007
Protein powder	Cardboard (Aluminum lined)	355	340	0.012	0.014
Mac and Cheese	Cardboard & Aluminum cheese packet	177	170	0.009	0.013
Egg	Cardboard	a	7	0.006	0.006
Oatmeal	Cardboard	532	510	0.015	0.025
Pasta	Cardboard	473	454	0.012	0.014
Rice	Cardboard	192	191	0.012	0.012
BBQ chips	Coated paper substrate	148	142	0.007	0.023
Butter	Coated paper substrate	473	454	ND	0.006
Pita chip	Coated paper substrate	217	208	0.010	0.030
Sea salt chips	Coated paper substrate	148	142	0.015	0.031
Apple juice	Glass	946	907	ND	0.003
Balsamic	Glass	236	227	0.003	0.005
Cinnamon	Glass	56	53	ND	ND
Honey	Glass	355	340	0.011	0.016
Jelly	Glass	488	468	0.006	0.009
Mayonnaise	Glass	473	454	0.005	0.009
Milk	Glass	946	980	0.003	0.004
Pasta sauce	Glass	769	737	0.012	0.016
Peanut butter	Glass	473	454	0.011	0.017
Pepper	Glass	68	65	0.010	0.018
Salsa	Glass	420	417	0.004	0.006
Salt	Glass	104	99	0.005	0.008
Seasoning	Glass	62	59	0.015	0.019
Yogurt	Glass	946	907	0.007	0.006
Bread	Plastic	799	765	0.010	0.014
Cereal	Plastic	355	340	0.014	0.023
Cookie	Plastic	192	184	0.011	0.016
Gelato	Plastic	473	387	0.005	0.006
Wafer Cookie	Plastic	266	225	0.011	0.019
Popcorn	Plastic	130	125	0.026	0.032
Tortilla (inside)	Plastic	319	306	0.013	0.021
Tortilla (outside)	Plastic	319	306	0.013	0.022
NON-ORGANIC FOODS					
33% Chocolate	Aluminum	95	90	0.012	0.014
Cheese	Plastic	a	7	0.005	0.007
Wafer Cookie	Plastic	325	311	0.008	0.021
Berry Energy Bar	Plastic	54	52	0.014	0.017
Chocolate Energy Bar	Plastic	54	52	0.016	0.020
Turkey	Plastic	a	7	0.012	0.014

ND, Non-detectable.

a Value not available.

After the baseline period, participants will be randomized (blocked by sex and ethnicity) to either Diet + BPA or Diet + No BPA, in a double-blinded fashion. The study statistician will computer generate the randomization scheme but will not have contact with any participant. During the treatment phase, both groups will be provided the same diet as the baseline phase, with the only difference being administration of BPA or placebo. Oral administration of BPA and placebo will occur on a wafer cookie, similar to our preliminary study and other previous pharmacokinetic studies [23,42]. For BPA, a single dosing solution (10 mg/ml) will be prepared by dissolving d6-BPA (C/D/N Isotopes, Pointe-Claire, Quebec) in absolute 95% ethanol (Acros Organics, Janssen Pharmaceuticalaan, Belgium). For placebo, d6-BPA will not be included in the ethanol solution. A research assistant not involved in any other aspect of this study will make the dosing solutions. Approximately 1 ml aliquots will be passed twice through a sterile micro filter to aid in removal of bacteria and placed onto a wafer cookie (20 g), allowing the ethanol to dry six to 8 h before daily consumption. The wafer cookies with BPA and placebo look, weigh, and taste identical. Participants will consume the wafer cookie each morning for 4 days. During the treatment phase, repeated assessment of 24-h urine collection for BPA concentrations will occur. In the morning on the 5th day, repeated assessment of fasting blood, skeletal muscle insulin sensitivity, hepatic glucose production, and fecal microbiome will occur. Administration of the final BPA or placebo (5 total doses) will occur 1 h prior to the hepatic glucose production and skeletal muscle insulin sensitivity measurement.

### 2.5 BPA dose selected and precautions

The dose of BPA selected in the study is consistent with studies evaluated by The European Food Safety Authority (EFSA) and the National Toxicology Program, that typical BPA exposures, based on occupational work, range from 0.43 to 100 μg/kg body weight [43,44]. The BPA dose selected is well below the FDA no-observed-adverse-effect level of 5 mg/kg BW per day [45], and is consistent with the US EPA safe dose [46]. To date, several experimental trials (including our previous pilot study) have administered BPA to humans with no observed gastrointestional distress, adverse effects, unintended participant harms, or other deleterious effects [23,24]. These experimental studies administered BPA at the US EPA defined “safe dose” of 50 μg⋅kg-BW^−1^, which is the same dose that will be used in the current study. In addition, several pharmacokinetics have administered 50–100 μg/kg BW of BPA with no reported side effects [42,[47], [48], [49]].

The current study has several precautions in place to minimize risk. Medical supervision (Dr. Ryan Hubbard) will occur throughout the entire study with daily blood pressure assessment and daily blood tests during treatment days that include liver, kidney, and immune functioning. A Comprehensive Chemistry Panel (CCP; liver and kidney functioning) and Complete Blood Count (CBC) test will be completed and evaluated by the medical doctor associated with the study. The CCP includes, glucose, blood urea nitrogen, creatinine, blood urea nitrogen/creatine, sodium, potassium, chloride, total carbon dioxide, calcium, total protein, albumin, globulin, albumin/globulin ratio, alkaline phosphatase, aspartate aminotransferase, alanine aminotransferase, total bilirubin, and glomerular filtration rate estimated. The CBC includes white blood cell, red blood cell, hemoglobin, hematocrit, mean corpuscular volume, platelet count. These tests will be done in “real time” and reviewed by the study medical doctor each day. Should any abnormally occur, the medical doctor will inform the investigators and the DSMB. The medical doctor specializing in internal medicine has the authority to cease participant treatment at any time and inform investigators and DSMB. If needed, the participant's physicians will be contacted to discuss treatment/assessment continuation for the participant. Also, all participants will be provided a 2-day “fresh food” diet after they have completed the study (or if drop out during the study). A “fresh food” diet has been shown to reduce urinary BPA concentrations by 66% [29], and we used a similar diet after our previous pilot study.

### 2.6 Urinary BPA and creatinine concentrations

At baseline and treatment periods, urine will be collected for 24-h [47], on consecutive days to minimize day-to-day variability of BPA [50]. After measuring urine volume with a graduated cylinder, 25 mL will be aliquotted into 5 separate BPA-free polypropylene tubes at each 6-h interval during collection and stored at −80 °C. All urine samples will be analyzed using established CDC protocol [51,52] using a high-performance liquid chromatography tandem mass spectrometry (LCMS/MS) as described previously [53]. The laboratory technician will be blinded to the identity of the samples and treatment allocation. To minimize the potential of sample contamination, analyses will be conducted in compliance with established Good Laboratory Practice methodological protocols [49,54,55] including 1) direct testing of all urine collection/storage apparatus (cannulae, tube holders, storage tubes) and storage blanks, 2) controls with high and low concentrations, and 3) replicate sample analysis. Limit of detection is 0.05 ng/ml.

### 2.7 BPA and BPS food analysis

Forty-five different, mainly organic, foods needed to feed participants were tested for BPA and BPS levels using a commercially available ELISA kits (Detroit R&D, Inc., Detroit MI; Table 1). Samples were prepared by either a liquid supernatant (n = 9 foods) or solid food (n = 36 foods) protocol, with BPA-HRP and BPS-HRP conjugates competing with the unknown concentration of BPA and BPS in food sample on a limited (and known) number of binding sites. Both protocols converted food samples into liquid states that could then be evaporated to obtain a concentrated sample with the BPA and BPS still intact. Each food sample was analyzed in triplicate and was assessed with a microplate photometer (Multiskan FC, Thermoscientific Inc., Waltham, MA) at an absorbance of 450 nm. Total binding and percent binding for each sample was calculated, and a 4 parameter sigmoidal standard curve was used to calculate concentrations of BPA and BPS in food.

### 2.8 Fasting hormones, hepatic glucose production and muscle insulin sensitivity

At baseline and treatment periods, fasting hormones, endocrine factors, and inflammatory markers linked to the pathogenesis of type 2 diabetes, including insulin, glucose, C-peptide, pro-insulin, adiponectin, 17-beta-estradiol, free fatty acids (FFA) and BPA concentration will be collected. Then a priming bolus of 200 mg [6,6-^2^H] glucose will be given, followed by a 90-min infusion of [6,6-^2^H] glucose at a rate of 2.5 mg/min by a peristaltic infusion pump (Harvard Apparatus Pump 22; Harvard Apparatus, Holliston, MA) to assess hepatic glucose production. Skeletal muscle insulin sensitivity (euglycemic hyperinsulinemic clamp technique) will then be assessed as others and we previously described [27,56,57]. Prior to insulin infusion, exhaled air will be collected for 15 min, using a ventilated hood and indirect calorimetry to determine basal substrate oxidation. Two infusions will be started using a peristaltic infusion pump: 1) primed (250 mU/m^−2^) constant infusion (40 mU/m^−2^•min^−1^) of insulin diluted in saline containing 3% (vol/vol^−1^) of the participants own blood; and 2) a variable infusion of 20% glucose saline solution with 2% spiked [6,6-^2^H] glucose, adjusted to maintain plasma glucose at 90 mg/dl, and continued for 120 min. Blood glucose analysis will occur every 5 min using the glucose oxidation method (GL5, Analox Instruments, Stourbridge, UK). Rates of glucose appearance (Ra) and glucose disposal (Rd) will be calculated using non-steady-state Steele equations [58]. Ra, which is comprised primarily of hepatic glucose production, will be averaged from t = −30 to t = 0 min. Rd, which reflect mostly skeletal muscle glucose uptake, will be averaged during the last 30 min and used to characterize skeletal muscle insulin sensitivity. Hepatic glucose production during the clamp will be calculated as the difference between Ra clamp and the exogenous glucose infusion rate. Hepatic glucose production will be used to estimate hepatic insulin sensitivity [57]. Glucose kinetics and clamp-derived carbohydrate oxidation will be determined by indirect calorimetry during the last 30 min of the clamp using standard equations [58]. Blood samples will be centrifuged at 4 °C for 15 min at 3000 g and then stored at −80 °C until subsequent analysis. Glucose isotopic enrichment will be measured by LCMS/MS, as we described previously [57]. Plasma insulin, C-peptide, pro-insulin, adiponectin, and 17-beta-estradiol will be measured using an ELISA assay (Millipore, Billerica, MA or Invitrogen Corporation Camarillo, CA). Plasma free fatty acids will be analyzed by a colorimetric assay (Wako Chemicals, Richmond, VA), and plasma BPA using LCMS/MS. Samples will be analyzed using Good Laboratory Methodological Protocols, including 1) analyzing each participants samples during the same laboratory run (lab technicians will be blinded to treatment), 2) urine and blood analyses will include a random sample for each subject blocked by treatment, 3) controls with high and low concentrations, 4) replicate samples, and 5) analysis repeated if coefficient of variation >7–10%.

### 2.9 Fecal microbiome

Fecal microbiome will be assessed by 16S rRNA gene sequencing for classification and relative quantitation of bacterial taxa. Fecal samples will be collected <1 h of defecation and DNA will be extracted from well-homogenized aliquots using industry-standard fecal DNA extraction kits. Polymerase chain reaction (PCR) will be used to amplify the V3–V4 variable regions of the 16 rRNA genes with 25 cycles of amplification to minimize PCR biases. PCR products will be sequenced in a paired-end protocol using the MiniSeq DNA sequencing platform. Sequence data will be run through a standard microbiome analysis pipeline. Bacterial diversity will be assessed at multiple taxonomic levels from Operational Taxonomic Units through Phylum, using observed counts as well as Simpson and Shannon diversity indices.

### 2.10 Statistical considerations

#### 2.10.1 Sample size calculation

Because there were no previous experimental studies over multiple days evaluating the direct effects of administration of BPA on skeletal muscle insulin sensitivity in humans, the power calculation and sample size for this study are based on the subject repeated measures of metabolized glucose in the original Defronzo and Matsuda euglycemic hyperinsulinemic clamp technique paper [59]. The standard deviation of the differences in repeated measures was found to be 0.45 mg/kg⋅min, which is equivalent to a medium effect size. With 40 total participants at baseline, assuming 10% lost to follow up at final assessment (N = 36) we have 80% power to detect a 0.52 glucose infusion rate (mg/kg-min) difference between Diet + BPA vs. Diet + No BPA, using an α = 0.05 and a 2-sided test of significance.

### 2.11 Statistical analysis

The goal in our analyses will be to compare participants randomized to Diet + BPA vs. Diet + No BPA. We conservatively expect 10% of participants will be lost to follow up. Statistical test will be conducted at the P < 0.05 level. Primary outcomes: A linear mixed effect model will be used to examine differences in skeletal muscle insulin sensitivity and hepatic glucose between groups adjusting for age, sex, hormonal contraceptives, education, income, physical activity levels, ethnicity/race, baseline BMI, baseline dietary intake and macronutrient intake. Exchangeable, autoregressive model (1), and unstructured covariance structures will be examined and conservatively selected using Bayesian information criterion. Secondary outcomes: Similarly, linear mixed effect models will be used to examine differences in hormones and changes in fecal microbiome community structure using the same covariates.

## 3 Discussion

Previous animal and human observational studies showed that BPA has negative health and weight consequences [[3], [4], [5],^7^,^12^,^15^,^19^,^27^,^30^,^41^,^52^]. Surprisingly, no published study to date has assessed whether BPA administration over several days negatively effects the pathogenesis of type 2 diabetes. The proposed randomized study is the first to examine the direct and potential causal effects of BPA administration, consistent with the US EPA safe dose, using gold standard measures over several days on skeletal muscle insulin sensitivity and hepatic glucose production; thus, this study will provide much needed randomized controlled experimental evidence as to whether BPA poses any health risk for type 2 diabetes.

Emergent data in humans have shown that single BPA administration immediately alters indices of glucose metabolism [23,24]. Specifically, we previously showed that oral BPA administration at the US EPA safe dose reduced glucose, insulin, and C-peptide concentrations in response to an oral glucose tolerance test [23]. This is corroborated by another recent study showing that BPA administration at the US EPA safe dose reduced insulin and C-peptide concentrations in response to glucose infusion [24]. Although these two human studies did not address potential mechanisms, data suggest that BPA has an effect on hepatic glucose production, skeletal muscle insulin sensitivity, and/or gastrointestinal absorption of glucose and fecal microbiome to reduce blood glucose, insulin, and C-peptide concentrations. Based in large part on these two previous published human studies, the current study was designed to assess potential mechanisms by which BPA is linked with type 2 diabetes, using gold standard measures of hepatic glucose production (via [6,6-^2^H] glucose infusion) and skeletal muscle insulin sensitivity (via euglycemic hyperinsulinemic clamp technique), and will explore the fecal microbiome.

Human exposure to BPA is extensive [52], and observation and cross-sectional studies have consistently showed that BPA exposure is related to negative health consequences with associations between urinary BPA concentrations and type 2 diabetes, pre-diabetes, insulin resistance, and hemoglobin A1c [[14], [15], [16],^26^,^41^]. Thus, the chronic influence of BPA exposure is currently observed through epidemiological studies, and the most important adverse effects of BPA appear to be caused by longer term, low dose exposure. In the current study 4 days of BPA exposure may be insufficient to alter hepatic glucose production and insulin sensitivity, but does not rule out potential adverse effects of longer exposure.

The study also has some limitations. The single BPA dose administered does not allow for examination of dose-response relationships. Also, participants of normal-weight were chosen given the potential confound of higher BPA exposure and insulin resistance with obesity [8] but the results may not be generalizable to other populations. This study will not assess directly assess insulin secretion, glucose absorption through the gastrointestional tract, cardiovascular disease risk markers or other pesticides, chemicals, or endocrine disruptors linked to type 2 diabetes (e.g. phthalates). Finally, the 5 doses of BPA exposure is not representative of longer-term exposure that the population experiences.

## 4 Conclusion

With emergent data showing that BPA has negative health and weight outcomes, it is important to understand the direct effects of BPA on human health. The proposed experimental study will examine whether BPA administration alters hepatic glucose production and skeletal muscle insulin sensitivity. Findings from this study provide a framework for future studies in this area, and provide the first, much needed experimental evidence using gold standard measures as to whether BPA consumption over several days poses any risk of type 2 diabetes.

## Funding support

American Diabetes Association 1-19-ICTS-044.

## Registered at ClinicalTrial.gov as identifier

NCT03771066.

## Disclosure summary

TH, HBG, AS, AS, CK, RH, SKM, MRL, KB have nothing to declare. SP has a grant form WW International unrelated to this work.
